# Effect of COVID-19 on Gynecologic Oncology Care: A Survey of Practicing Gynecologic Radiation Oncologists in the United States

**DOI:** 10.1016/j.adro.2023.101188

**Published:** 2023-02-26

**Authors:** Jeffrey V. Brower, Sylvia S. Rhodes, Jill S. Remick, Andrea L. Russo, Emily F. Dunn, Diandra N. Ayala-Peacock, Daniel G. Petereit, Kristin A. Bradley, Neil K. Taunk

**Affiliations:** a Department of Human Oncology, University of Wisconsin School of Medicine and Public Health, Madison, Wisconsin; b Radiation Oncology Associates–New England, Manchester, New Hampshire; c Radiation Oncology, University of Pennsylvania Perelman School of Medicine, Philadelphia, Pennsylvania; d Department of Radiation Oncology, Winship Cancer Institute, Emory School of Medicine, Atlanta, Georgia; e Department of Radiation Oncology, Massachusetts General Hospital, Boston, Massachusetts; f Department of Radiation Oncology, Willamette Valley Cancer Institute and Research Center, Eugene, Oregon; g Department of Radiation Oncology, Duke Women's Health, Raleigh, North Carolina; h Department of Radiation Oncology, Monument Health Cancer Care Institute, Rapid City, South Dakota

## Abstract

**Purpose:**

The COVID-19 pandemic has placed demands and limitations on the delivery of health care. We sought to assess the effect of COVID-19 on the delivery of gynecologic oncologic care from the perspective of practicing radiation oncologists in the United States.

**Methods and Materials:**

An anonymous online survey was created and distributed to preidentified radiation oncologists in the United States with clinical expertise in the management of gynecologic patients. The survey consisted of demographic questions followed by directed questions to assess specific patterns of care related to the COVID-19 pandemic.

**Results:**

A total of 47 of 96 invited radiation oncologists responded to the survey for a response rate of 49%. Fifty-six percent of respondents reported an increase in locally advanced cervical cancer with no similar increase for endometrial, vulvar, or vaginal patients. Most respondents (66%) reported a pause in surgical management, with a duration of 1 to 3 months being most common (61%). There was a reported increased use of shorter brachytherapy regimens during the pandemic. Most providers (61%) reported caring for at least 1 patient with a positive COVID-19 test. A pause or delay in treatment due to COVID-19 positivity was reported by 45% of respondents, with 55% reporting that patients chose to delay their own care because of COVID-19–related concerns. Total treatment times >8 weeks for patients with cervical cancer were observed by 33% of respondents, but occurred in >25% of patients.

**Conclusions:**

Data from this prospectively collected anonymous survey of practice patterns among radiation oncologists reveal that the COVID-19 pandemic resulted in delays initiating care, truncated brachytherapy treatment courses, and a reported increase in locally advanced cervical cancer cases at presentation. These data can be used as a means of self-assessment to ensure appropriate decision making for gynecologic patients during the endemic phase of COVID-19.

## Introduction

The COVID-19 pandemic placed unprecedented, extraordinary demands and constraints on health care systems. During the pandemic, health care resources were prioritized to save the most lives and maximize improvements in patients’ length of life.^1^^,^^2^ To conserve valuable resources and minimize disease transmission, routine hospital and health care services were disrupted, including routine health maintenance as well as nonemergent and elective surgeries.^3^, ^4^, ^5^, ^6^, ^7^, ^8^ Consequently, cancer screening, referral of symptomatic patients, diagnosis, and definitive treatments were hindered or delayed.^9^, ^10^, ^11^ Patients with limited access to telemedicine may have been disproportionately affected by the disruption in routine medical care and cancer screening.

Patients with gynecologic cancers, including cervical, endometrial, vaginal, and vulvar cancers, may have been particularly affected by the COVID-19 pandemic due to their complex clinical management and socioeconomical factors. For example, one study conducted in 6 New York City hospitals found the fatality rate in gynecologic oncology patients with a COVID-19 infection to be 14.0%, while another study showed more than one-third of gynecologic cancer patients in New York City experienced treatment delay, change, or cancellation during the first 2 months of the pandemic.^12^^,^^13^

Patients with cancer are often more vulnerable to COVID-19 infections and severe complications due to their underlying illness and often immunosuppressed status.^14^, ^15^, ^16^ They have experienced cancellation or postponement of scheduled appointments, cancer treatment, and operations due to fears of going to the hospital, mandated quarantine for active infection, resources being prioritized for those seriously ill with COVID-19, or guideline recommendations to minimize immune compromise.^10^^,^^17^, ^18^, ^19^, ^20^ Many cancer centers used nonstandard approaches such as neoadjuvant chemotherapy, radiation therapy, or hormonal therapy while surgeries were being delayed. However, these difficult decisions involved risk of disease progression or emergent complications.^10^ The American Society of Clinical Oncology, European Society for Medical Oncology, and the American Brachytherapy Society and other brachytherapy experts published several guidelines to inform systemic cancer treatment and guidelines to outline delaying, abbreviating, or omitting radiation therapy where appropriate.^21^, ^22^, ^23^, ^24^, ^25^

Gynecologic cancers frequently require radiation treatment, yet many radiation oncology clinics saw a large decline in patient volume during the pandemic due to voluntary or mandated rollbacks in treatment volume to limit patient exposure.^26^^,^^27^ It is well established that delays in timely completion of chemoradiation and brachytherapy can negatively affect oncologic outcomes in cancer of the cervix.^25^ However, limited data exist on the perceived effect of the pandemic on gynecologic cancer treatment. In this study, we performed a survey of practicing radiation oncologists to assess how the COVID-19 pandemic has affected radiation therapy in gynecologic cancer treatment.

## Methods and Materials

An anonymous online survey was created using Qualtrics (SAP, Provo, UT), an online platform for the creation, distribution, and tracking of surveys and responses. The survey consisted of a section of demographic questions followed by directed questions to assess specific patterns of care related to the COVID-19 pandemic within practitioners’ respective practices. The survey consisted of 67 questions and was designed using a multistep process, during which appropriate revisions were made by a preselected group of practicing academic and community radiation oncologists with expertise in gynecologic care across various career stages, geographic locations, and practice settings.

The survey was distributed to preidentified United States radiation oncologists with clinical expertise in the management of gynecologic patients based upon NRG Oncology, American Society for Radiation Oncology, and American Brachytherapy Society participation or clinical scope of practice. These represent individuals actively engaged in the discussion of research related to and clinical care of patients with gynecologic malignancies. In addition to the individuals included from NRG oncology etc, American Society for Radiation Oncology, and American Brachytherapy Society lists, the authors provided emails for individuals known to participate in the delivery of gynecologic care within radiation oncology. Responses were recorded and analyzed using the Qualtrics platform. The survey remained open for 5 weeks (October 18 to November 29, 2021) and 2 reminder emails were sent. There was no financial incentive for completing the survey. The study was deemed exempt by our institutional review board in accordance with 45 Code of Federal Regulations Part 46. Descriptive statistics were used for data interpretation and exported using the Qualtrics platform.

## Results

### Demographics

Forty-seven of 96 invited radiation oncologists with expertise in the management of patients with gynecologic cancer responded to the survey for a response rate of 49%. Demographic data of the respondents are summarized in Table 1. Most respondents (66%) characterized their practices as academic and within an urban setting. Seventy percent of respondents cared for patients at a designated comprehensive cancer center. All geographic regions were represented, and there was a roughly equal distribution of responses from male (42.6%) and female (55.3%) radiation oncologists. Only 17% of respondents had participated in formal brachytherapy fellowships. Most respondents were >5 years out from the completion of training, and only 17% were <5 years into independent practice.

**Table 1:** Demographic characteristics of respondents (N = 47)

	Respondents	
Characteristic	%	No.
Sex		
Male	42.6	20
Female	55.3	26
Nonbinary	0.0	0
Prefer not to answer	2.1	1
Practice type		
Academic	66.0	31
Private practice	34.0	16
Government	0.0	0
Practice community setting		
Urban	66.0	31
Suburban	29.8	14
Rural	4.3	2
Designated cancer center		
Yes	70.2	33
No	29.8	14
Geographic location		
Northeast	27.7	13
Southeast	21.3	10
Midwest	14.9	7
Southeast	10.6	5
West	25.5	12
Years in practice postresidency		
<5	17	8
5-10	29.8	14
10-15	27.7	13
>15	25.5	12
Fellowship trained in brachytherapy		
Yes	17	8
No	83	39

### Pause in care and telemedicine

During the pandemic, 66% of respondents reported a pause in surgical management of patients with gynecologic cancers at their institution. For most respondents, the delay in surgical management was 1 to 3 months (61%) with a minority (<29%) reporting a <1 month delay (Table 2). Twenty-eight percent of radiation oncologists reported a complete transition to telemedicine for new patient visits, most commonly for a duration of 1 to 3 months (54%) or <1 month (31%). Similarly, only 32% reported a complete switch to telemedicine for follow-up visits, for which the duration was 1 to 3 months in 47% and <1 month in 40%.

**Table 2:** Pause in care and telemedicine visits for gynecologic patients during the pandemic

	Respondents	
Question	%	No.
Pause in surgical management		
Yes	66.0	31
No	34.0	16
Duration of pause in surgical management		
<1 mo	29.0	9
1-3 mo	61.3	19
3-6 mo	3.2	1
>6 mo	6.5	2
Complete switch to telemedicine for new patient visits		
Yes	27.7	13
No	72.3	34
Hybrid	0.0	0
Duration of complete switch to telemedicine for new patient visits		
<1 mo	30.8	4
1-3 mo	53.8	7
3-6 mo	15.4	2
>6 mo	0.0	0
Complete switch to telemedicine for follow-up visits		
Yes	31.9	15
No	68.1	32
Hybrid	0.0	0
Duration of complete switch to telemedicine for follow-up visits		
<1 mo	40.0	6
1-3 mo	46.7	7
3-6 mo	6.7	1
>6 mo	6.7	1

### Observed increase in locally advanced gynecologic cancer

During the pandemic, 56% of respondents reported a subjective increase in patients presenting with locally advanced cervical cancer in comparison to prepandemic presentation (Table 3). There was no similar observed increase in advanced stage endometrial or vaginal/vulvar cancer during the pandemic, with 82% of respondents reporting no change in the presentation of these patients.

**Table 3:** Observed increase in locally advanced cancer

	Respondents	
Cancer type	%	No.
Cervical cancer		
Yes	55.6	25
No	44.4	20
Endometrial cancer		
Yes	18.2	8
No	81.8	36
Vaginal or vulvar cancer		
Yes	18.2	8
No	81.8	36

### Radiation fractionation before and during the pandemic

Before the COVID-19 pandemic, among respondents, the most commonly used brachytherapy regimens in the curative intent treatment of cervical cancer were 5.5 Gy × 5, 6 Gy × 5, and 7 Gy × 4 (Table 4). During the pandemic, these remained the most common regimens in the setting of curative intent brachytherapy for cervical cancer; however, there was an increased use of 7 Gy × 4 during the pandemic (33% vs 49% before and during, respectively). For adjuvant brachytherapy in the management of endometrial cancer the most common regimens were 5.5 Gy × 4, 6 Gy × 5, and 7 Gy × 3 being the most commonly used regimens both before and during the pandemic. There was, however, an increased utilization of 7 Gy × 3 during the pandemic (51% vs 35%).

**Table 4:** Fractionation used before and during the pandemic

	Respondents			
	Before		During	
Fractionation	%	No.	%	No.
Curative intent brachytherapy for cervical cancer				
5.5 Gy × 5	23.3	10	11.6	5
6 Gy × 5	32.6	14	23.3	10
7 Gy × 4	32.6	14	48.8	21
8 Gy × 3	9.3	4	14.0	6
Other	2.3	1	2.3	1
Adjuvant vaginal cuff brachytherapy for endometrial cancer				
5.5 Gy × 4	18.6	8	16.3	7
6 Gy × 5	41.9	18	25.6	11
7 Gy × 3	34.9	15	51.2	22
Other	4.7	2	7.0	3

### Effect on clinical care

Sixty-five percent of respondents reported caring for at least 1 patient with a positive COVID-19 test during the pandemic. Sixty-five percent reported institutional protocol of testing for COVID-19 before initiation of treatment (14% before external beam radiation therapy [EBRT], 25% before brachytherapy, and 25% before anesthesia use only). All respondents reported that <25% of patients’ care was delayed by COVID-19 testing. Sixty percent of providers reported continuing radiation treatments during COVID-19 positivity (19.5% EBRT alone, 34% EBRT + brachytherapy) (Table 5). A pause or delay in treatment due to COVID-19 positivity was reported in 45% of respondents. Sixteen percent reported having at least 1 patient die while undergoing radiation treatment as a result of COVID-19–related complications. Fifty-five percent of respondents reported that patients chose to delay their own care because of COVID-19–related concerns; however, 91% reported that this occurred in <25% of their patients. Total treatment times of >8 weeks for patients with cervical cancer were observed by 33% of providers, but they reported that this occurred in <25% of patients (Table 5).

**Table 5:** COVID-19’s effect on gynecologic patients

	Respondents	
Question	%	No.
Positive COVID-19 test during treatment		
Yes	65.1	28
No	34.9	15
Active treatment of patients with COVID-19		
No	39.0	16
Yes, EBRT only	19.5	8
Yes, brachytherapy only	7.3	3
Yes, EBRT and brachytherapy	34.1	14
Pause or delay in treatment due to positive COVID-19 test		
Yes	45.2	19
No	54.8	23
Died due to COVID-19 or related causes		
Yes	16.3	7
No	83.7	36
Total treatment time >8 wk for cervical cancer		
Yes, <25% of patients	32.6	14
Yes, 25%-50% of patients	0.0	0
Yes, >50% of patients	0.0	0
No	67.4	29
Required COVID-19 test before treatment		
Yes	65.1	28
No	34.9	15
When is COVID-19 test required?		
Before EBRT	14.3	4
Before brachytherapy	25.0	7
Before EBRT and brachytherapy	35.7	10
Before anesthesia only	25.0	7
Delayed by testing		
<25% of patients	100.0	28
25%-50% of patients	0.0	0
>50% of patients	0.0	0
Did patients self-delay due to COVID-19 risk?		
Yes	55.8	24
No	44.2	19
Self-delay		
<25% of patients	91.7	22
25%-50% of patients	8.3	2
>50% of patients	0.0	0

*Abbreviation:* EBRT = external beam radiation therapy.

## Discussion

The COVID-19 pandemic has and will continue to have far-reaching effects on social interaction, global economies, and health care delivery. In addition to strains placed on the health care industry as a direct result of COVID-19–related medical care, the management of unrelated medical conditions have been affected indirectly. Cancer care has been significantly affected, with the long-term implications of such anticipated to be appreciated for years to come, particularly with respect to stage migration, increased early mortality, delay in cancer-related research, and decreased screening.^28^, ^29^, ^30^, ^31^, ^32^, ^33^ Similar to other areas of medicine, gynecologic oncology care has been affected due to both patient- and provider-related factors.^34^^,^^35^

Recognizing the potential effects of COVID-19 on the delivery of gynecologic oncology care, key stakeholder groups formulated recommendations to maximize radiation oncology care during the pandemic.^24^^,^^25^ Expert consensus statements focused on the prioritization of patient care based on tiered categorization of cancer severity, emphasis on curative intent therapies, appropriate timing of treatment, use of hypofractionation, and appropriateness of palliation.^24^ Similarly, recommendations regarding gynecologic brachytherapy were devised and included the importance of timely treatments, adoption of shortened fractionation regimens, and options for the delivery of temporizing therapies in the event that brachytherapy could not be immediately delivered as a result of clinic-related and COVID-19–related limitations.^25^

Several investigators performed survey-based analyses of COVID-19’s effect on gynecologic care, focusing on the perspective of gynecologic oncologists.^36^^,^^37^ The Society of Gynecologic Oncology surveyed its members to assess the effects of COVID-19 on the delivery of gynecologic care.^38^ They reported a decrease in surgical and clinical productivity with 83% of respondents experiencing a ≥50% reduction in surgical volume. Seventy-two percent reported using more neoadjuvant chemotherapy. Telehealth care dramatically increased, with >50% of Society of Gynecologic Oncology respondents completing >75% of visits via telemedicine. Clinical trials were also dramatically affected, with 61% reporting that they stopped enrolling patients on trials. In addition to clinical concerns, COVID-19 affects the treating physician as well, with only 25% reporting no compromise on their individual well-being during the pandemic.

In our survey, we assessed the effect on the radiation management of gynecologic malignancies during the COVID-19 pandemic. A significant number of respondents reported experiencing a pause in surgical management; however, for most, it lasted only 1 to 3 months. In addition, a minority reported a complete transition to telemedicine during the pandemic. Greater than 50% of respondents reported an increase in presentations of locally advanced cervical cancer but did not observe this for other gynecologic malignancies. Most respondents reported the delay in seeking care to be due to patients’ COVID-19–related fears. Despite more than half of respondents reporting caring for at least 1 patient with a positive COVID-19 test during the pandemic, most respondents reported completing EBRT and brachytherapy for patients with cervical cancer within 8 weeks. The increased use of shorter brachytherapy regimens during the pandemic likely helped to avoid prolongation of overall treatment time.

These findings suggest that the COVID-19 pandemic has affected the timely delivery of care for gynecologic patients from a radiation oncology standpoint. Most notably, providers experienced delays in care as a result of pauses in surgical management, a subjective increase in locally advanced presentations of cervical cancer, and a transition to shorter courses for brachytherapy delivery. It is anticipated that delays in care as a result of COVID-19 as well as ongoing patient fears may have contributed to the increase in locally advanced cervical cancer patients. In addition, while not surveyed, the transition to shorter courses for brachytherapy may have been 2-fold: to expedite care that may have already been delayed and to reduce COVID-19 exposure for both patients and providers during the pandemic. The transition to shorter courses for brachytherapy included well-established fractionation regimens and is not anticipated to affect the oncologic or toxicity-related outcomes of treatment.

There are a number of limitations to the current study. While the 49% response rate is relatively high for this type of survey study, it still only represents responses from a limited number of practicing radiation oncologists. Despite an attempt to obtain responses from a relatively equal amount of academic and private practice radiation oncologists, most responses in this study were obtained from academic radiation oncologists, limiting the generalizability of these results. In addition, as with most questionnaire-based survey studies, there can be subjectivity in responses.

## Conclusion

As the global effect of the COVID-19 pandemic continues to be present in all aspects of life, the effect on clinical care is an ongoing concern and will evolve over time. An awareness of the issues is critical to inform efforts to minimize the effect on cancer diagnosis and treatments. Furthermore, as we shift into a maintenance phase of the pandemic, COVID-19–related, clinical care delivery limitations may have an increasing effect on physician burnout. This will likely further contribute to adequacy of delivery of care concerns.^39^^,^^40^

These data represent an assessment of the effects of COVID-19 on gynecologic care, specifically within radiation oncology. These data can be used as a means of ongoing self-assessment to ensure that the adequacy of clinical care is minimally affected and to inform future discussions about resource allocation.
